# Melatonin Ameliorates Stress-Induced Oocyte Developmental Impairment: Evidence from In Vivo and In Vitro Models

**DOI:** 10.3390/ani16213403

**Published:** 2026-10-28

**Authors:** Chaoqun Cong, Junyu Wang, Yaqi Zhou, Xuedi Bian, Fuyin Fu, Mingjiu Luo, Shuai Gong

**Affiliations:** Shandong Provincial Key Laboratory for Livestock Germplasm Innovation & Utilization, College of Animal Science and Technology, Shandong Agricultural University, Tai’an 271018, China; allen9778v@163.com (C.C.); 13370685817@163.com (J.W.); 15275999891@163.com (Y.Z.); 13287037783@163.com (X.B.); 15288916369@163.com (F.F.); luomj@sdau.edu.cn (M.L.)

**Keywords:** melatonin, EGCG, stress, oocyte developmental competence, oxidative stress

## Abstract

Stress compromises oocyte quality and reproductive performance, yet the comparative efficacy of MT and other antioxidants under stress remains unclear. This study compared MT and EGCG using an in vivo mouse restraint stress model and an in vitro porcine oocyte maturation model. In stressed mice, MT (0.1, 1, and 10 mg/kg) and EGCG (35, 65, and 100 mg/kg), administered via intraperitoneal (i.p.) injection every 12 h during the 48 h restraint period, improved oocyte developmental rates at appropriate doses, with MT showing superior efficacy at the optimal dose (1 mg/kg). Notably, MT, but not EGCG, reduced serum cortisol and CRH levels in stressed mice. In porcine oocytes, CRH supplementation during 48 h in vitro maturation (IVM) impaired oocyte maturation and embryo development, and MT dose-dependently rescued these defects, accompanied by reduced reactive oxygen species (ROS) accumulation and decreased cumulus cell apoptosis. RT-PCR analysis revealed that porcine oocytes and cumulus cells expressed *MT1*, but not *MT2*, transcripts. Collectively, evidence from two complementary models (mouse in vivo and porcine in vitro) supports MT as a promising supplement for oocyte protection under stress, through systemic and cellular mechanisms demonstrated in different species.

## Simple Summary

Stress adversely affects oocyte quality and female fertility through elevated stress hormones and oxidative damage. In this study, we used two complementary stress models to investigate the protective effects of melatonin (MT) and epigallocatechin gallate (EGCG) on oocyte developmental competence: an in vivo mouse restraint stress model and an in vitro porcine oocyte stress model induced by the stress hormone corticotropin-releasing hormone (CRH). Consistent results from both models demonstrated that MT improved oocyte and embryo development more effectively than EGCG. In the mouse in vivo model, MT, but not EGCG, decreased serum concentrations of cortisol and CRH in stressed animals. In the porcine in vitro model, MT directly ameliorated CRH-triggered oocyte injury by mitigating oxidative stress and cumulus cell apoptosis. RT-PCR further showed that porcine oocytes and cumulus cells express the *MT1* receptor subtype. Concordant findings from the two models support that MT serves as a promising supplement to preserve oocyte quality under stress, with prospective uses in assisted reproduction and livestock breeding.

## 1. Introduction

Stress, whether psychological, physiological, or environmental, represents a significant threat to female reproductive health and fertility. In both humans and livestock, exposure to stressors activates the hypothalamic–pituitary–adrenal (HPA) axis, leading to elevated circulating levels of corticotropin-releasing hormone (CRH), adrenocorticotropic hormone, and glucocorticoids such as cortisol and corticosterone [1,2]. These stress mediators can directly disrupt ovarian function and compromise oocyte developmental competence [3]. The restraint stress model in mice has been widely used to investigate stress-induced reproductive impairment. Previous studies demonstrated that 48 h restraint stress significantly elevates serum CRH and glucocorticoid levels, induces oxidative stress in ovarian granulosa cells and oocytes, and reduces oocyte maturation and subsequent embryonic development [4]. CRH, in particular, has been shown to directly induce apoptosis of ovarian cells and impair oocyte developmental potential, making it a key mediator of stress-related reproductive damage. In porcine oocytes, we have previously demonstrated that CRH exposure during in vitro maturation (IVM) directly impairs oocyte competence by inducing cumulus cell apoptosis through CRH receptor 1 [5].

Accumulating evidence from restraint stress and other psychological stress models indicates that oxidative stress is a primary mechanism underlying stress-induced oocyte damage [6,7,8]. Under stress conditions, excessive reactive oxygen species (ROS) are generated in granulosa cells and oocytes, and the imbalance between ROS production and antioxidant defense mechanisms leads to ROS accumulation [9]. Excessive ROS can induce oxidative damage to critical cellular components, including mitochondrial DNA, proteins, and lipids, thereby disrupting mitochondrial function, impairing cytoskeletal integrity, and interfering with intracellular signaling pathways involved in meiotic maturation [10]. These adverse effects ultimately result in reduced oocyte maturation rate, compromised fertilization capacity, and diminished embryonic developmental potential [11,12].

Melatonin (*N*-acetyl-5-methoxytryptamine, MT), a multifunctional molecule primarily secreted by the pineal gland, possesses unique properties that distinguish it from conventional antioxidants [13]. Beyond its potent direct free radical scavenging activity, MT acts through high-affinity G protein-coupled membrane receptors, designated MT1 (MTNR1A) and MT2 (MTNR1B), to regulate downstream signaling pathways involved in cell survival, redox homeostasis, and energy metabolism [14,15]. MT has been shown to protect oocytes from various insults, including chronic stress-induced meiotic defects [16], heat stress, postovulatory aging, and toxin exposure [17,18,19]. Building on these findings, the present study investigated whether MT protects oocytes from stress-induced developmental impairment using both in vivo and in vitro models.

In contrast, epigallocatechin gallate (EGCG), the major catechin in green tea, is a polyphenolic antioxidant with well-documented radical scavenging and metal-chelating properties [20]. We and others have previously shown that maternal injection of EGCG protects mouse oocytes from restraint stress-induced oxidative damage and improves blastocyst formation and postimplantation development [21,22], indicating that EGCG is an appropriate reference antioxidant for comparison with MT. However, EGCG has relatively poor bioavailability, limited membrane permeability, and potential pro-oxidant effects at high concentrations, which may restrict its efficacy [23]. Therefore, EGCG was included as a reference antioxidant to compare with MT in the present study.

Although antioxidants have been shown to improve oocyte quality under various conditions, their protective effects against stress-induced oocyte damage remain insufficiently characterized. In particular, it is unclear whether melatonin (MT) and other antioxidants differ in their ability to mitigate stress-mediated oocyte impairment, and the underlying mechanisms remain to be elucidated. Therefore, the present study investigated the protective effects of MT against stress-induced oocyte developmental impairment using both an in vivo mouse restraint stress model and an in vitro porcine oocyte IVM model supplemented with corticotropin-releasing hormone (CRH), with epigallocatechin gallate (EGCG) as a reference antioxidant. 

The novelty of this study includes (1) a direct head-to-head comparison of MT and EGCG in a physiological in vivo restraint-stress model; (2) a multi-species, multi-level experimental strategy that links systemic endocrine stress responses to cellular antioxidant and anti-apoptotic mechanisms; and (3) identification of MT1 receptor expression in porcine oocytes and cumulus cells as a potential local target of MT action. Collectively, these findings provide a theoretical basis for the application of MT in mitigating stress-induced reproductive impairment in assisted reproduction and livestock breeding.

## 2. Materials and Methods

All female mice employed in the present work were obtained from Shandong Pengyue Laboratory Animal Technology Co., Ltd. (Jinan, China). Porcine ovaries were sourced from the Feicheng Slaughterhouse of Yinbao Food Corporation Ltd. (Tai’an, China). All animal-related experimental procedures were approved by the Animal Care and Use Committee of Shandong Agricultural University on 28 February 2023 (Permit No. SDAUA-2023-020). Chemicals and reagents were predominantly sourced from Sigma-Aldrich Corp. (St. Louis, MO, USA), except where otherwise indicated.

### 2.1. Mouse Restraint Stress Model and Drug Administration

Mice of the Kunming strain (8–10 weeks old) were kept in a room with a 14:10 light-dark cycle with the dark phase starting at 20:00 h, with free access to food and water. The restraint-stress protocol was performed as previously described with minor modifications [24,25]. The restraint apparatus consisted of custom-made fine wire-mesh micro-cages (10 cm × 2.5 cm × 2 cm, length × width × height) with access doors at both ends. Each micro-cage was fitted with a drinking nozzle and a food compartment to allow free access to water and food, and was placed inside a standard home cage to ensure consistent lighting and ambient temperature with the non-stressed control group. Within each micro-cage, mice could move back and forth freely but could not turn around; this setup limited overall body movement without producing physical injury to the animals. Notably, a key procedural modification was made to reduce confounding stress from repeated handling: all intraperitoneal injections during the 48 h restraint period were administered through the mesh openings of the cages, without removing the mice from the restraint apparatus. Melatonin (MT, Cat. No. M5250, Sigma-Aldrich, St. Louis, MO, USA) was dissolved in a small volume of 1% ethanol and then diluted with sterile physiological saline to obtain working solutions. Epigallocatechin gallate (EGCG, Zhejiang Yixin Pharmaceutical Co., Ltd., Lanxi, China) was dissolved in sterile saline containing the same final concentration of ethanol (1%, *v*/*v*) as the MT working solutions. The vehicle solution for control injections consisted of sterile saline containing the same low final concentration of ethanol as present in MT working solutions. Briefly, following intraperitoneal (i.p.) injection of 10 IU equine chorionic gonadotropin (eCG) to stimulate follicular development, female mice in stress groups were individually placed inside ventilated fine wire-mesh restraint cages for continuous 48 h restraint; this wire-mesh-based apparatus limited animal movement without producing physical injury to mice. During the 48 h restraint period, MT (0.1, 1, and 10 mg/kg body weight) or EGCG (35, 65, and 100 mg/kg body weight) was administered via i.p. injection every 12 h. The MT doses were selected based on the effective dose ranges reported in previous animal studies [26,27], and the EGCG doses followed our previously established maternal injection protocol in restraint-stressed mice [21]. Mice were randomly assigned to four main experimental groups: (1) Non-stressed control group: mice housed in standard home cages without restraint, receiving intraperitoneal (i.p.) injection of vehicle solution every 12 h; (2) Restraint stress group: mice subjected to 48 h restraint stress, receiving i.p. injection of vehicle solution every 12 h; (3) Restraint + MT groups: mice subjected to 48 h restraint stress, receiving i.p. injection of MT at three dose levels (0.1, 1, and 10 mg/kg body weight) every 12 h; (4) Restraint + EGCG groups: mice subjected to 48 h restraint stress, receiving i.p. injection of EGCG at three dose levels (35, 65, and 100 mg/kg body weight) every 12 h. All injections during the restraint period were administered through the mesh openings of the restraint cages without removing the mice from the apparatus, to minimize additional confounding stress caused by repeated handling. Each experimental group contained 6–10 mice, and all experiments were independently replicated at least three times. Each treatment was repeated with 25–30 oocytes per replicate, and the total number of oocytes used in each group was 298 (control), 336 (stress), 156 (MT 0.1 mg/kg), 163 (MT 1 mg/kg), 178 (MT 10 mg/kg), 126 (EGCG 35 mg/kg), 133 (EGCG 65 mg/kg), and 112 (EGCG 100 mg/kg), respectively.

### 2.2. Mouse Oocyte Collection, In Vitro Maturation, and Parthenogenetic Activation

After 48 h of restraint stress (corresponding to 48 h after eCG injection), mice were euthanized by cervical dislocation, and ovaries were immediately collected and placed in pre-warmed M2 medium. Germinal vesicle (GV)-stage cumulus-oocyte complexes (COCs) were recovered by puncturing the large antral follicles on the ovary in pre-warmed M2 medium (37.5 °C) under an SMZ745T stereomicroscope (Nikon, Tokyo, Japan) at magnifications ranging from 10× to 40×; the GV stage was confirmed by the presence of a clearly visible germinal vesicle (Supplementary Figure S1A). Only COCs containing more than three layers of unexpanded cumulus cells and an oocyte of at least 75 μm in diameter with homogeneous cytoplasm were selected for subsequent culture, as described in our previous study [5].

Collected COCs were washed three times and cultured in groups of 25–30 in 100 μL droplets of maturation medium covered with mineral oil. The maturation medium consisted of TCM-199 (Gibco, Grand Island, NY, USA) supplemented with 10% (*v*/*v*) fetal calf serum (FCS), 1 mg/mL 17β-estradiol, 24.2 mg/L sodium pyruvate, 0.05 IU/mL FSH, 0.05 IU/mL luteinizing hormone, and 10 ng/mL epidermal growth factor. COCs were cultured at 37.5 °C in a humidified atmosphere of 5% CO_2_ in air for 24 h. After IVM, cumulus cells were removed by gentle pipetting in M2 medium containing 0.1% (*w*/*v*) hyaluronidase. Oocytes with a visible first polar body (PB1) were considered mature (MII stage) and selected for parthenogenetic activation (PA).

MII oocytes were activated by incubation for 6 h in Ca^2+^-free CZB medium containing 10 mM strontium chloride (SrCl_2_) and 5 μg/mL cytochalasin B at 37.5 °C under 5% CO_2_ in humidified air. The Ca^2+^-free CZB medium was prepared in-house with the following components per 100 mL of ultrapure water: NaCl (0.4770 g), KCl (0.0360 g), KH_2_PO_4_ (0.0161 g), MgSO_4_ (0.0144 g), NaHCO_3_ (0.2110 g), sodium lactate (0.3507 g), sodium pyruvate (0.0030 g), EDTA (0.0041 g), L-glutamine (0.0146 g), BSA (0.5000 g), penicillin (0.0060 g), and streptomycin sulfate (0.0050 g), and was filter-sterilized through a 0.22 μm membrane filter before use. At the end of activation, oocytes containing one or two well-developed pronuclei were considered successfully activated. Activated oocytes were then cultured in 100 μL droplets of CZB medium covered with mineral oil at 37.5 °C under 5% CO_2_ in humidified air. When embryos developed beyond the 3- to 4-cell stage, 5.5 mM glucose was added to the CZB medium. Developmental rates were assessed at 24 h (cleavage/2-cell), 48 h (4-cell), and 96 h (blastocyst) after activation. The 2-cell rate was calculated as the percentage of 2-cell embryos relative to activated oocytes; the 4-cell rate was calculated as the percentage of 4-cell embryos relative to 2-cell stage embryos; and the blastocyst rate was calculated as the percentage of blastocysts relative to 4-cell stage embryos.

### 2.3. Serum Stress Hormone Measurement

At the end of the 48 h restraint period, mice were decapitated rapidly, and trunk blood (approximately 1 mL) was collected into ice-cooled centrifugal tubes and centrifuged at 1700× *g* for 10 min at 4 °C to separate serum. Serum samples were stored at −80 °C until hormone assay. Serum cortisol concentration was measured by radioimmunoassay (RIA) conducted by the Central Hospital of Tai-An City using commercial kits from 3V Biomedical Techniques Co., Ltd. (Weifang, China), as described in our previous study [25]. The kit measures total cortisol in serum, including cortisol bound to corticosteroid-binding globulin (CBG). The minimum detection limit was 0.15 ng/mL, and the intra- and inter-assay coefficients of variation were both less than 10%. The cross-reactivity of the cortisol RIA kit with corticosterone was 0.11% (tested at 50% binding). The validity of cortisol measurement in mice by RIA has been established in our previous study, which characterized the dynamics and correlation of serum cortisol and corticosterone under different physiological and stressful conditions in mice [25]. CRH concentration was measured using a commercially available competitive enzyme-linked immunosorbent assay (ELISA) kit (Mouse CRH/CRF ELISA Kit, Catalog No. E03P0031, BlueGene, Shanghai, China) according to the manufacturer’s instructions. The detection range was 10–1000 pg/mL with a minimum sensitivity of 9.2 pg/mL. All samples were assayed in duplicate. Serum samples were collected from 4 to 6 mice per group, and each sample was assayed in duplicate. The hormone measurements for each group were based on 4–6 biological replicates.

### 2.4. Porcine Oocyte Collection and IVM with CRH and MT Treatment

Porcine ovaries were collected from 6-month-old pigs and transported to the laboratory within 2 h in sterile physiological saline (30–35 °C) supplemented with 100 IU/mL penicillin and 0.05 mg/mL streptomycin. Cumulus-oocyte complexes (COCs) were recovered by aspirating 3–6 mm follicles using a syringe containing Dulbecco’s phosphate-buffered saline (D-PBS, HyClone, Logan, UT, USA) supplemented with 0.88 mM CaCl_2_·2H_2_O, 0.49 mM MgCl_2_·6H_2_O, 0.1% polyvinyl alcohol, 0.03 mM phenol red, 50 IU/mL penicillin, and 0.05 mg/mL streptomycin. Recovered COCs were washed three times in the same D-PBS solution, and only those with uniform ooplasm and surrounded by 3–5 layers of compact cumulus cells were selected to ensure consistent oocyte quality across groups for subsequent IVM.

Oocyte maturation was performed in TCM-199 medium (Gibco, Grand Island, NY, USA) supplemented with 0.91 mM sodium pyruvate, 4.0 mg/mL BSA, 3.05 mM D-glucose, 50 IU/mL penicillin, 0.05 mg/mL streptomycin, 0.05 IU/mL FSH, 0.05 IU/mL LH, 10 ng/mL EGF, and 0.57 mM cysteine. Maturation medium was aliquoted into 96-well plates (150 μL per well), overlaid with 100 μL mineral oil, and pre-equilibrated at 38.5 °C under 5% CO_2_ in humidified air for at least 3 h before use. Approximately 25 COCs per well were transferred into the pre-equilibrated wells and cultured for 48 h under the same conditions. To establish the in vitro stress model, CRH (C3042, Sigma-Aldrich, St. Louis, MO, USA) was dissolved in sterile water to prepare a stock solution, stored in aliquots at −20 °C, and diluted to the working concentration immediately before use. CRH was then added to the IVM medium at a final concentration of 1 × 10^−6^ M, following the protocol established in our previous study [5]. To evaluate the protective effects of MT, MT was dissolved in anhydrous ethanol to prepare a 1 × 10^−2^ M stock solution, which was serially diluted to 1 × 10^−4^ M and 1 × 10^−6^ M working solutions (each 1000-fold concentrated relative to the corresponding final concentration). MT working solutions were added to the CRH-containing IVM medium at 1‰ (*v*/*v*) to achieve final concentrations of 1 × 10^−9^, 1 × 10^−7^, and 1 × 10^−5^ M. These concentrations were selected based on previous porcine oocyte IVM studies [28] and further optimized by gradient concentration screening in the present study. The control group was supplemented with an equal volume of sterile water and anhydrous ethanol (1‰ each, *v*/*v*) to match the vehicle concentrations across all groups. Oocyte maturation rate was calculated as the percentage of MII oocytes relative to total cultured oocytes. Following maturation culture, cumulus cells were removed by vortexing oocytes in D-PBS containing 0.1% (*w*/*v*) hyaluronidase for 3 min at 38.5 °C, and the resulting denuded oocytes (DOs) were collected. At least six independent biological replicates were included for each treatment to ensure statistical validity (*n* = 176–202 COCs per group). Each treatment was repeated 6–8 times with approximately 25 COCs per replicate, and the total number of oocytes used in each group was 176 (control), 202 (CRH), 186 (CRH + 1 × 10^−9^ M MT), 191 (CRH + 1 × 10^−7^ M MT), and 197 (CRH + 1 × 10^−5^ M MT), respectively.

### 2.5. Porcine Oocyte Parthenogenetic Activation and Embryo Development

Mature denuded oocytes were first treated with 5 μM ionomycin contained in D-PBS for 5 min in the dark. Then, the oocytes were washed three times in porcine zygote medium-3 (PZM-3) and incubated for 5 h in PZM-3 containing 2 mM 6-DMAP (12–16 oocytes per drop). After removal of 6-DMAP, the activated oocytes were transferred into mineral-oil-covered PZM-3 (12–16 oocytes per drop) and cultured at 38.5 °C under 5% CO_2_ in humidified air for 7 days [29]. Cleavage, 4-cell, and blastocyst rates were evaluated at 24 h, 48 h, and 168 h post-parthenogenetic activation, respectively. Cleavage rate was the percentage of 2-cell embryos relative to total matured oocytes; the 4-cell embryo rate was the percentage of 4-cell embryos relative to total cleaved embryos; the blastocyst rate was the percentage of blastocysts relative to total 4-cell embryos.

### 2.6. Detection of ROS

Intra-oocyte reactive oxygen species (ROS) levels were measured using 10 μM 2′,7′-dichlorodihydrofluorescein diacetate (DCFH-DA, HY-D0940, MCE, Monmouth Junction, NJ, USA) [3]. Oocytes were incubated with the probe at 37 °C for 10 min in the dark, washed three times with D-PBS, and immediately observed and imaged using a fluorescence microscope. Fluorescence signals were detected at an excitation wavelength of 488 nm, and all images were captured with fixed microscope parameters to ensure data consistency. Fluorescence intensity of individual oocytes was analyzed using ImageJ software (version 1.53t, National Institutes of Health, Bethesda, MD, USA) and expressed as average fluorescence intensity. Each treatment was repeated at least three times with approximately 25 oocytes per replicate, and the total number of oocytes analyzed per group was 60–83.

### 2.7. Hoechst Staining for Cumulus Cell Apoptosis

Hoechst staining was used to assess cumulus cell (CC) apoptosis, as previous studies have demonstrated that Hoechst staining and TUNEL are comparable methods for detecting apoptosis [30]. After 48 h of IVM, COCs from each treatment group were treated with 0.1% hyaluronidase in D-PBS to remove CCs. The CCs from 30 to 40 COCs were collected into a 1.5 mL centrifuge tube and centrifuged at 200× *g* for 5 min at room temperature to obtain a CC precipitate. The supernatant was discarded, and 200 μL of PBS containing 0.01 mg/mL Hoechst 33342 was added to the CC precipitate. The cell precipitate was gently pipetted to evenly disperse the CCs, incubated in the dark for 5 min, and then centrifuged at 200× *g* for 5 min at room temperature. The supernatant was discarded, and 5 μL of PBS was added to the precipitate to resuspend the CCs. Then, 5 μL of the CC suspension was placed in the center of a slide, covered with a coverslip, and observed under a Leica DMLB fluorescence microscope (Leica Microsystems, Wetzlar, Germany) with a 40× objective. Six to eight fields containing approximately 200 CCs were randomly selected, and the number of apoptotic CCs was counted. Apoptotic cells were identified by pyknotic nuclei filled with condensed heterochromatin, whereas healthy cells showed normal nuclei with sparse heterochromatin spots [31]. The apoptotic rate was calculated as the percentage of apoptotic CCs relative to the total number of CCs counted. Each treatment group was assessed in at least three independent biological replicates, and the total number of cumulus cells scored per group was 1584 (control), 1483 (CRH), and 1318 (CRH + MT), respectively.

### 2.8. RT-PCR Analysis of MT1 and MT2 Receptor Expression

Real-time PCR was performed as previously described [32]. Total RNA was extracted from denuded oocytes (DOs), cumulus cells (CCs), and mural granulosa cells (MGCs) using TRIzol reagent (Invitrogen, Carlsbad, CA, USA) according to the manufacturer’s instructions. For each biological replicate, 200 DOs were collected after IVM, and the corresponding CCs were obtained from the same 200 oocytes following hyaluronidase treatment (0.1% hyaluronidase in D-PBS). Mural granulosa cells (MGCs) were isolated from the follicular fluid of 3–6 mm antral follicles. Briefly, follicular fluid was aspirated from individual follicles using a syringe fitted with a 21-gauge (green) needle (0.8 mm outer diameter) and transferred into 50 mL centrifuge tubes. The tubes were placed in a 37 °C water bath for 5 min of natural sedimentation, after which the supernatant was carefully discarded. The cell sediment was washed three times with Dulbecco’s phosphate-buffered saline (D-PBS) by resuspension and gentle sedimentation. Under a stereomicroscope, only sheet-like aggregates of granulosa cells—morphologically characteristic of MGCs lining the follicular wall—were selectively collected using a fine mouth pipette, while intact cumulus-oocyte complexes (COCs), single erythrocytes, and cell debris were discarded (see Figure S2 for representative morphological features of MGC sheets and COCs). The collected MGC aggregates were then washed an additional three times with D-PBS to remove residual contaminating cells. Cells from 5 to 10 follicles were pooled for each biological replicate. After extraction, RNA was resuspended in diethyl pyrocarbonate-treated MilliQ water (DEPC-dH_2_O) and digested with RNase-free DNase I (Takara Bio, Dalian, China) to eliminate genomic DNA contamination. RNA concentration was measured spectrophotometrically at 260 nm, and RNA purity and integrity were assessed by the A260:A280 ratio (1.8–2.0) and electrophoresis on a 1% agarose gel.

Reverse transcription was performed using Superscript III Reverse Transcriptase (Invitrogen, Carlsbad, CA, USA) in a total volume of 20 μL. Briefly, (i) 1 μL of RNA sample, 1 μL of Oligo dT_18_ (Takara Bio, Dalian, China), and 11 μL of DEPC-dH_2_O were mixed in a 0.2 mL reaction tube; (ii) the mixture was incubated at 65 °C for 10 min in a thermal cycler (Thermo Scientific, Hudson, NH, USA); (iii) the reaction tube was cooled on ice for 2 min followed by a brief centrifugation (200× *g*, 4 °C); (iv) 4 μL of 5× reverse transcription buffer, 0.5 μL of RNase inhibitor, 2 μL of dNTP, and 0.5 μL of Superscript III Reverse Transcriptase were added to the reaction tube; and (v) the mixture was incubated at 55 °C for 30 min, followed by 85 °C for 5 min, and stored at −20 °C until use.

PCR amplification was performed using gene-specific primers for porcine MT1, MT2, tumor necrosis factor-α (TNF-α), and the housekeeping gene β-actin (primer sequences are listed in Table 1). The PCR reaction mixture (25 μL total volume) consisted of 2 μL of cDNA template, 12.5 μL of 2× PCR Master Mix, 1 μL of each forward and reverse primer (10 μM), and 8.5 μL of nuclease-free water. The amplification conditions were initial denaturation at 94 °C for 5 min, followed by 35 cycles of denaturation at 94 °C for 30 s, annealing at 60 °C for 30 s, extension at 72 °C for 30 s, and a final extension at 72 °C for 10 min. PCR products were separated by electrophoresis on a 1.5% agarose gel containing ethidium bromide and visualized under ultraviolet light. The presence or absence of target bands was recorded. β-actin served as an internal loading control, and TNF-α served as a positive control to confirm cDNA quality and successful reverse transcription. At least three independent biological replicates were performed for each cell type.

### 2.9. Statistical Analysis

All statistical analyses were performed using SPSS software (version 22.0, SPSS Inc., Chicago, IL, USA) and GraphPad Prism software (version 6.0, GraphPad Software, San Diego, CA, USA). Data are presented as mean ± standard error of the mean (SEM) from at least three independent replicates. Differences among multiple groups were analyzed by one-way analysis of variance (ANOVA) followed by Tukey’s post hoc test for pairwise comparisons. Differences were considered statistically significant at *p* < 0.05.

## 3. Results

### 3.1. Dose-Dependent Effects of MT and EGCG on Oocyte Developmental Competence in Restraint-Stressed Mice

**Figure 1:**
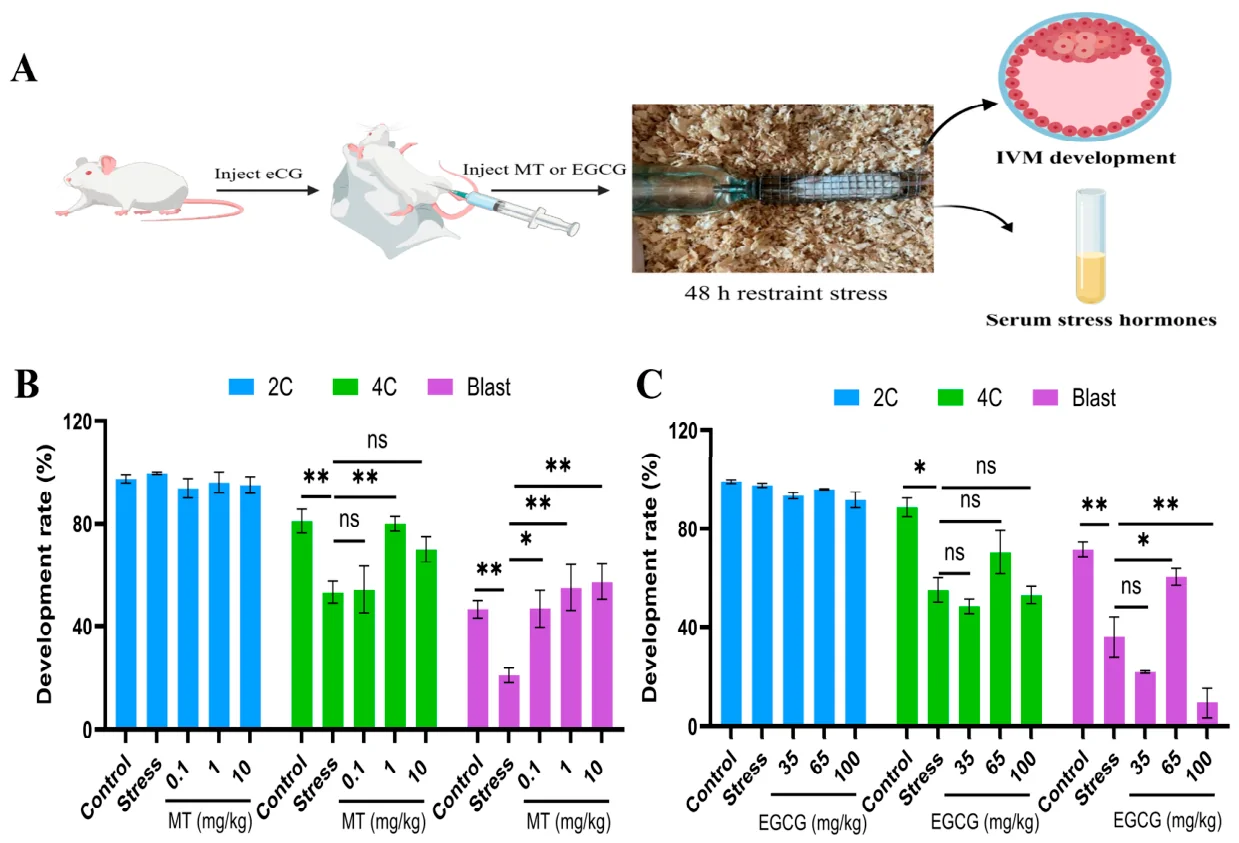
Melatonin improves oocyte developmental competence in restraint-stressed mice more effectively than EGCG. (**A**) Schematic diagram of the experimental design. Female mice were intraperitoneally (i.p.) injected with 10 IU eCG to stimulate follicular development, and melatonin (MT, 0.1, 1, and 10 mg/kg) or EGCG (35, 65, and 100 mg/kg) was administered via i.p. injection every 12 h during the 48 h restraint stress period; oocytes were subsequently collected for in vitro maturation (IVM) and embryo development assessment. (**B**) Developmental rates of 2-cell (2C), 4-cell (4C), and blastocyst (Blast) stage embryos in the control, stress, and MT-treated groups (0.1, 1, and 10 mg/kg). (**C**) Developmental rates of 2-cell (2C), 4-cell (4C), and blastocyst (Blast) stage embryos in the control, stress, and EGCG-treated groups (35, 65, and 100 mg/kg). Data are presented as the mean ± SEM of at least three independent experiments. * *p* < 0.05, ** *p* < 0.01, ns (not significant) indicates no statistically significant difference.

To evaluate and compare the protective effects of MT and EGCG on oocyte developmental competence under stress conditions, dose–response experiments were first conducted using the mouse restraint stress model (Figure 1A). Forty-eight hours of restraint stress significantly reduced the 4-cell rate (53.2 ± 4.4% vs. 81.1 ± 4.7%, *p* = 0.002) and the blastocyst rate (20.9 ± 2.8% vs. 46.5 ± 3.4%, *p* = 0.004) compared with the non-stressed control, whereas the 2-cell rate was not significantly affected (99.6 ± 0.4% vs. 97.4 ± 1.6%). These results confirmed the successful establishment of the stress-induced oocyte damage model.

MT administration during the restraint period improved oocyte developmental rates (Figure 1B). At 0.1 mg/kg, MT significantly increased the blastocyst rate (46.9 ± 7.3% vs. 20.9 ± 2.8% in the stress group, *p* = 0.02) but did not improve the 4-cell rate (54.3 ± 9.1%). At 1 mg/kg, MT exerted the most pronounced protective effect, restoring the 4-cell rate to 80.0 ± 2.9% (*p* = 0.003 vs. stress), a level comparable to that of the non-stressed control, and increasing the blastocyst rate to 55.0 ± 9.0% (*p* = 0.007 vs. stress), which exceeded that of the control group. At 10 mg/kg, MT maintained the highest blastocyst rate (57.3 ± 6.5%, *p* = 0.004 vs. stress), although the 4-cell rate (69.9 ± 4.9%) was not fully restored (*p* = 0.052). These results indicate that MT protects oocyte developmental competence across a broad dose range, with 1 mg/kg being the optimal dose.

EGCG also affected oocyte development in a dose-dependent manner, but with a narrower effective range than MT (Figure 1C). At 35 mg/kg, EGCG did not significantly improve the 4-cell (48.31 ± 2.89%) or blastocyst (21.74 ± 0.51%) rates compared with the stress group (*p* > 0.05). At 65 mg/kg, EGCG significantly increased the blastocyst rate (60.45 ± 3.35% vs. 36.08 ± 11.51%, *p* = 0.021) and partially restored the 4-cell rate (70.49 ± 8.78%), although the latter did not reach statistical significance (*p* = 0.08). In contrast, a high dose of EGCG (100 mg/kg) markedly reduced the blastocyst rate to 9.25 ± 5.96% (*p* = 0.009 vs. stress), indicating dose-limiting toxicity at high concentrations. Collectively, MT protected oocytes from stress-induced developmental impairment across a broader effective dose range and with greater consistency than EGCG.

### 3.2. MT, but Not EGCG, Attenuates Stress Hormone Secretion at the Optimal Dose

**Figure 2:**
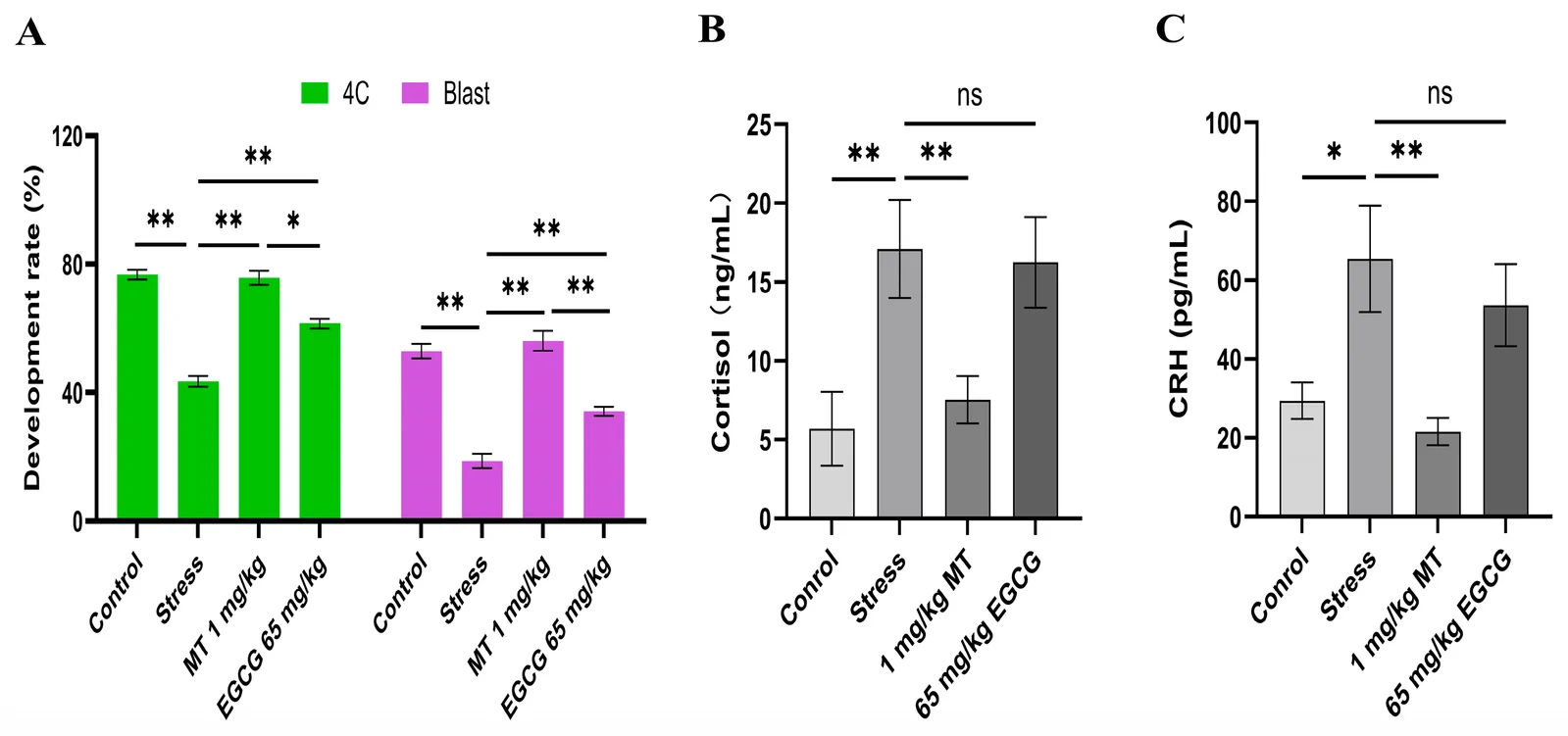
Effects of optimal-dose melatonin and EGCG on oocyte developmental competence and serum stress hormones in restraint-stressed mice. Based on the dose-screening results (Figure 1), mice were subjected to 48 h restraint stress and injected intraperitoneally with the optimal dose of melatonin (MT, 1 mg/kg) or EGCG (65 mg/kg) every 12 h; a non-stressed control group and a stress-only group were included. (**A**) Developmental rates of 4-cell (4C) and blastocyst (Blast) stage embryos derived from oocytes of control, stress, MT-treated, and EGCG-treated mice. (**B**) Serum cortisol levels (ng/mL) in control, stress, MT-treated, and EGCG-treated mice. (**C**) Serum corticotropin-releasing hormone (CRH) levels (pg/mL) in control, stress, MT-treated, and EGCG-treated mice. Data are presented as the mean ± SEM of at least three independent experiments. * *p* < 0.05, ** *p* < 0.01, ns (not significant) indicates no statistically significant difference.

To verify the dose-screening results and investigate whether the superior efficacy of MT could be attributed to its unique ability to modulate the systemic stress response, mice were treated with the optimal dose of MT (1 mg/kg) or EGCG (65 mg/kg) during the 48 h restraint period (Figure 2A). Consistent with the screening experiments, restraint stress significantly reduced the 4-cell (43.55 ± 1.73% vs. 76.78 ± 1.51%) and blastocyst (18.71 ± 2.19% vs. 52.96 ± 2.34%) rates. MT treatment fully restored the 4-cell (75.79 ± 2.19%) and blastocyst (56.19 ± 3.09%) rates to levels comparable to those of the non-stressed control, whereas EGCG only partially restored the 4-cell (61.51 ± 1.54%) and blastocyst (34.21 ± 1.46%) rates, which remained significantly lower than those of the control group. The 2-cell rate was not significantly affected by any treatment.

Serum stress hormone levels were then measured in the same batch of mice (Figure 2B,C). Restraint stress significantly elevated serum cortisol (17.102 ± 1.558 ng/mL vs. 5.69 ± 1.165 ng/mL) and CRH (65.43 ± 6.029 pg/mL vs. 29.391 ± 2.061 pg/mL) levels compared with the non-stressed control. MT administration significantly reduced serum cortisol (7.535 ± 0.756 ng/mL) and CRH (21.572 ± 1.734 pg/mL) levels in stressed mice, bringing them to levels comparable to (and for CRH, slightly below) those of the non-stressed control. In contrast, EGCG administration had no significant effect on either serum cortisol (16.24 ± 1.439 ng/mL) or CRH (53.648 ± 4.644 pg/mL) levels, which remained significantly elevated relative to the control. These findings reveal a critical distinction between the two antioxidants: MT possesses the unique ability to attenuate the systemic stress response by reducing stress hormone secretion, whereas EGCG acts primarily through direct antioxidant effects without modulating the HPA axis.

### 3.3. Direct Effects of Melatonin and EGCG on Developmental Competence of Oocytes from Restraint-Stressed Mice During In Vitro Maturation

**Figure 3:**
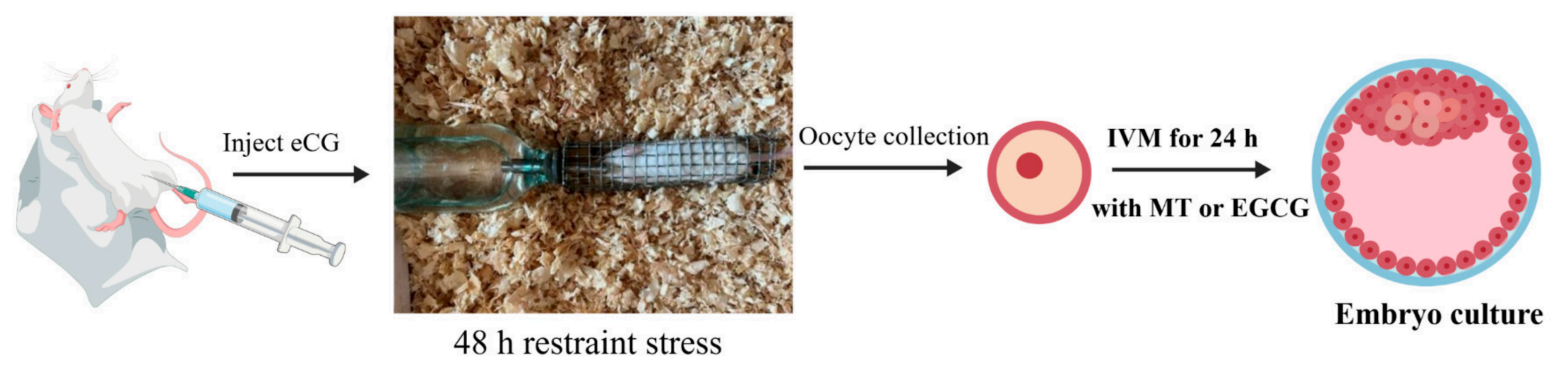
Schematic diagram of the experimental design for evaluating the direct effects of melatonin and EGCG on oocytes from restraint-stressed mice during in vitro maturation.

To evaluate the direct cellular effects of MT and EGCG independent of systemic endocrine modulation, GV-stage oocytes were collected from mice after 48 h restraint stress and treated with MT or EGCG during IVM (Figure 3). In this ex vivo system, antioxidants were present only during in vitro culture, excluding their systemic effects on the HPA axis. As shown in Table 2, restraint stress significantly reduced the 4-cell rate (58.2 ± 2.5% vs. 78.9 ± 1.4%, *p* < 0.01) and blastocyst rate (20.8 ± 2.1% vs. 44.9 ± 1.9%, *p* < 0.01) compared with the control, confirming that the stress imprint persisted in collected oocytes.

MT supplementation during IVM improved development in a dose-dependent manner. At 1 × 10^−5^ M, MT significantly increased both the 4-cell rate (71.2 ± 7.2%, *p* < 0.05) and the blastocyst rate (46.9 ± 7.3%, *p* < 0.01) compared with the restraint group, restoring the blastocyst rate to the control level. EGCG at 20 μg/mL significantly increased the 4-cell rate (72.2 ± 6.6%, *p* < 0.05) but failed to improve the blastocyst rate (28.5 ± 5.1%, *p* > 0.05). Thus, MT improved development through the blastocyst stage, whereas EGCG only improved the 4-cell rate. These findings indicate that MT provides more complete direct cellular protection than EGCG, and prompted us to further investigate MT’s protective mechanism using an in vitro porcine CRH stress model.

### 3.4. MT Dose-Dependently Alleviates CRH-Induced Damage in Porcine Oocytes During IVM

**Figure 4:**
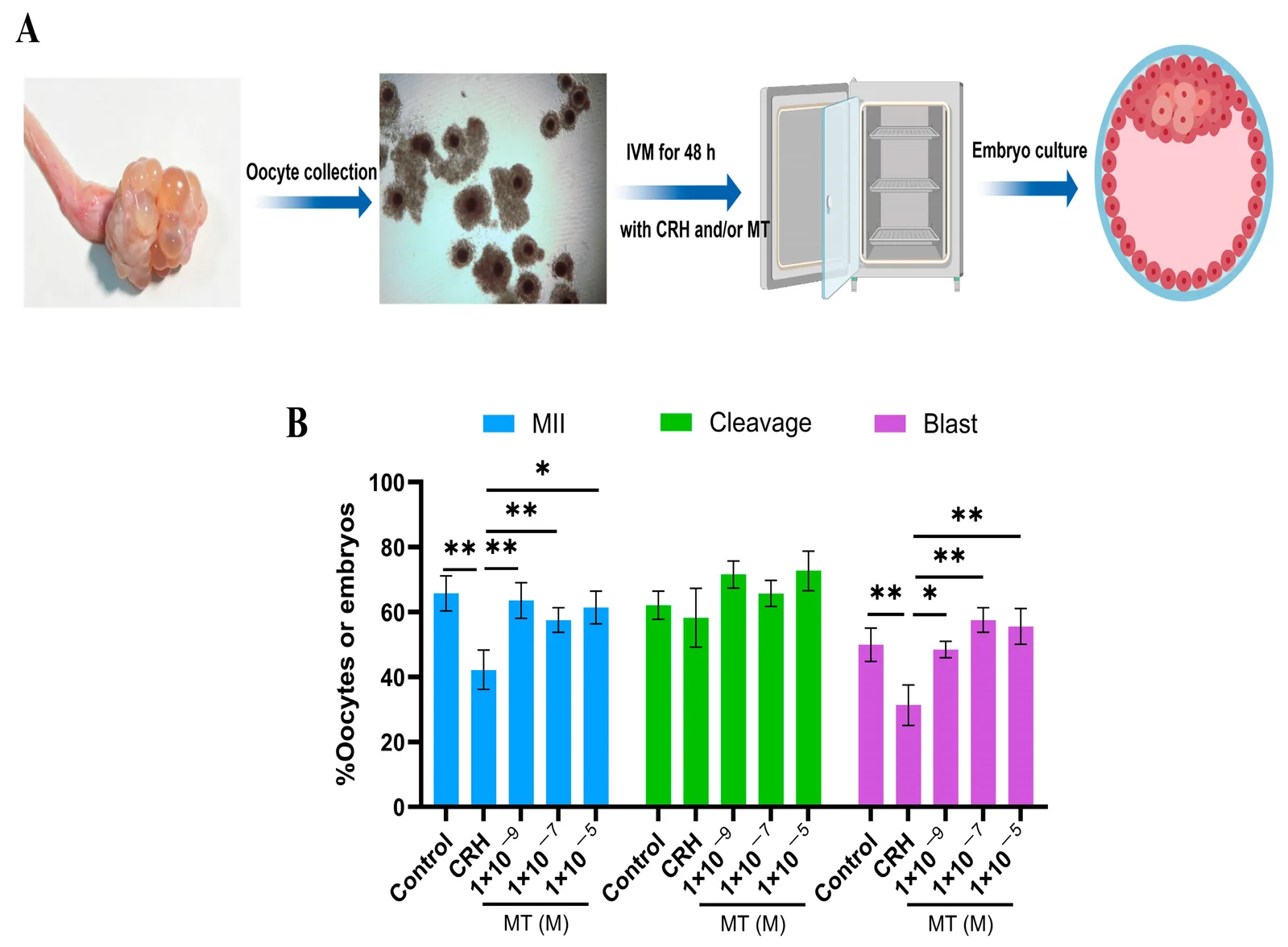
Melatonin dose-dependently alleviates CRH-induced impairment of porcine oocyte maturation and subsequent embryo development in an in vitro stress model. (**A**) Schematic diagram of the experimental workflow. Porcine ovaries were collected, cumulus-oocyte complexes (COCs) were retrieved by follicular aspiration, and oocytes were subjected to in vitro maturation (IVM) for 48 h in medium supplemented with corticotropin-releasing hormone (CRH) and/or melatonin (MT), followed by parthenogenetic activation and embryo culture. (**B**) Rates of MII oocytes, cleavage, and blastocyst formation in the control, CRH-treated, and CRH plus MT-treated groups (MT at 1 × 10^−9^, 1 × 10^−7^, and 1 × 10^−5^ M). Data are presented as the mean ± SEM of at least three independent experiments. * *p* < 0.05, ** *p* < 0.01.

To investigate the direct protective mechanism of MT at the cellular level, independent of systemic effects, an in vitro porcine oocyte maturation model was employed in which CRH was added to the IVM medium to simulate stress conditions (Figure 4A). CRH supplementation at 1 × 10^−6^ M significantly reduced the MII maturation rate (42.24 ± 6.04% vs. 65.77 ± 5.36%, *p* = 0.002) and the blastocyst rate (31.39 ± 6.26% vs. 49.95 ± 5.17%, *p* = 0.01) compared with the untreated control, consistent with our previous report of CRH’s direct deleterious effects on porcine oocyte competence [5], whereas the cleavage rate was not significantly affected (58.25 ± 9.02% vs. 62.12 ± 4.34%, *p* = 0.643).

Concurrent supplementation of MT during IVM significantly rescued CRH-impaired oocyte development at all three concentrations tested (Figure 4B). At 1 × 10^−9^ M, MT restored the MII rate to 63.58 ± 5.49% (*p* = 0.006 vs. CRH) and the blastocyst rate to 48.48 ± 2.48% (*p* = 0.016 vs. CRH), both comparable to the untreated control. At 1 × 10^−7^ M, MT increased the MII rate to 61.86 ± 3.75% (*p* = 0.01 vs. CRH) and produced the highest blastocyst rate among all groups (57.52 ± 3.85%, *p* = 0.0004 vs. CRH). At 1 × 10^−5^ M, MT maintained the MII rate (61.46 ± 4.99%, *p* = 0.012 vs. CRH) and the blastocyst rate (55.59 ± 5.51%, *p* = 0.001 vs. CRH). The cleavage rate was not significantly altered by MT supplementation at any concentration (71.57 ± 4.21%, 65.75 ± 3.96%, and 72.68 ± 6.10% for 1 × 10^−9^, 1 × 10^−7^, and 1 × 10^−5^ M, respectively; all *p* > 0.05). Considering the overall developmental performance, 1 × 10^−7^ M was identified as the optimal concentration of MT, which not only restored the MII rate but also yielded the highest blastocyst rate, and this concentration was therefore used in the subsequent mechanism experiments.

### 3.5. MT Reduces Oxidative Stress and Cumulus Cell Apoptosis in CRH-Treated Porcine COCs

**Figure 5:**
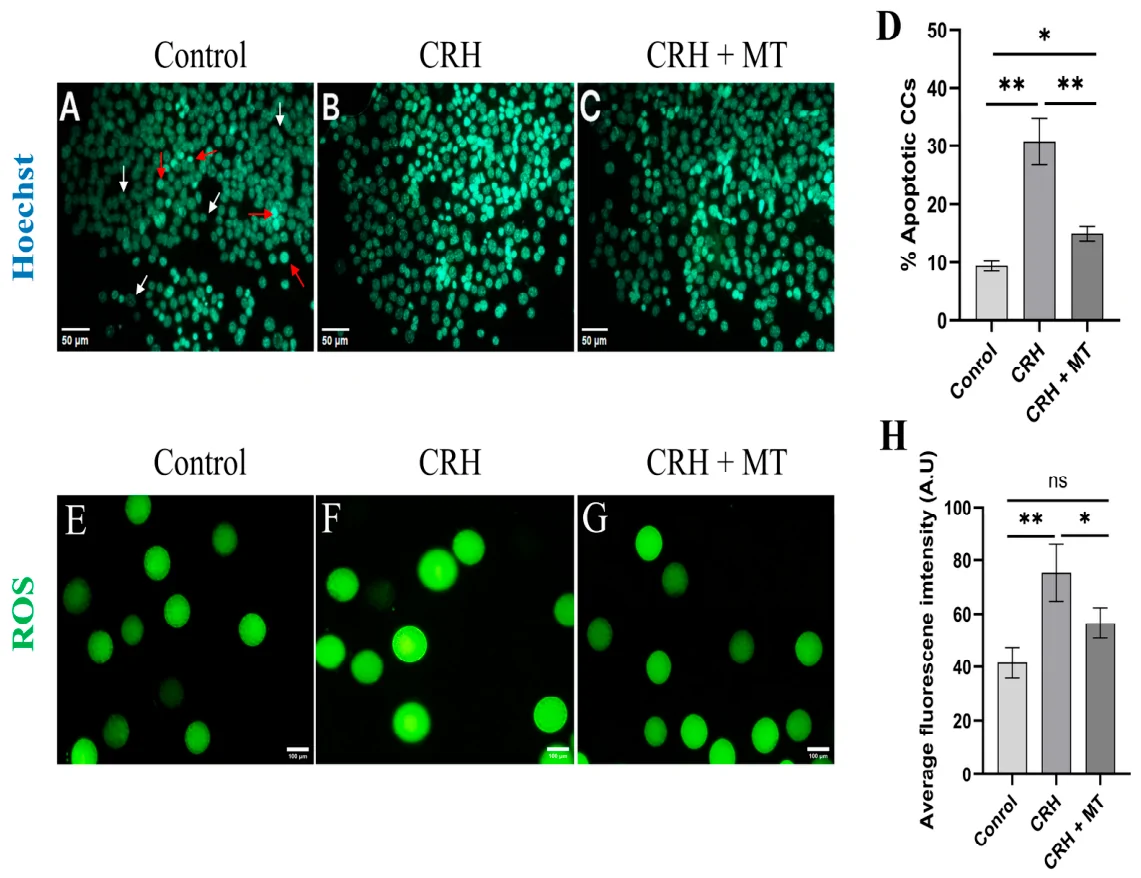
Melatonin alleviates CRH-induced cumulus cell apoptosis and oxidative stress in porcine oocytes. Porcine cumulus-oocyte complexes were matured in vitro for 48 h under control, CRH-treated, or CRH plus MT-treated conditions. (**A**–**C**) Representative fluorescence images of Hoechst 33342 staining showing cumulus cell apoptosis in the control (**A**), CRH-treated (**B**), and CRH + MT-treated (**C**) groups. Red arrows indicate apoptotic cumulus cells characterized by pyknotic, highly condensed nuclei with strongly enhanced Hoechst fluorescence; white arrows indicate normal cumulus cells with intact nuclei and evenly distributed chromatin. Scale bar = 50 μm. (**D**) Quantification of the apoptotic cumulus cell (CC) rate. (**E**–**G**) Representative fluorescence images of DCFH-DA staining showing intracellular ROS levels in MII oocytes in the control (**E**), CRH-treated (**F**), and CRH + MT-treated (**G**) groups. (**H**) Quantification of the average fluorescence intensity in arbitrary units (A.U.) reflecting intracellular ROS levels. Data are presented as the mean ± SEM of at least three independent experiments. * *p* < 0.05, ** *p* < 0.01, ns (not significant) indicates no statistically significant difference.

To elucidate the cellular mechanisms underlying MT’s direct protective effects, intracellular ROS levels and cumulus cell apoptosis were evaluated in porcine oocytes and cumulus cells matured under CRH-induced stress with or without MT supplementation (1 × 10^−7^ M; Figure 5).

Hoechst 33342 staining was performed to visualize the nuclear morphology of cumulus cells (Figure 5A–C). In the control group, most cumulus cells displayed normal nuclei with sparse heterochromatin spots (Figure 5A). After CRH treatment, numerous cumulus cells exhibited pyknotic nuclei filled with condensed heterochromatin, a hallmark of apoptosis (Figure 5B). Notably, concurrent MT supplementation markedly reduced the number of apoptotic cumulus cells (Figure 5C). Quantitative analysis showed that CRH treatment significantly increased the apoptotic cumulus cell (CC) rate compared with the control (30.75 ± 2.13% vs. 9.39 ± 0.69%, *p* < 0.01; Figure 5D). MT supplementation significantly decreased the apoptotic CC rate in CRH-treated COCs (14.92 ± 0.71%, *p* < 0.05 vs. CRH; Figure 5D), although it remained slightly higher than that of the control.

Intracellular ROS levels in MII oocytes were further assessed by DCFH-DA staining (Figure 5E–G). Control oocytes exhibited weak green fluorescence (Figure 5E), whereas CRH-treated oocytes displayed markedly enhanced fluorescence (Figure 5F). Consistent with the cumulus cell apoptosis results, CRH treatment significantly elevated the average fluorescence intensity, reflecting increased intracellular ROS accumulation (75.0 ± 6.08 vs. 41.0 ± 3.51 A.U., *p* < 0.01; Figure 5H). MT supplementation significantly reduced ROS accumulation in CRH-treated oocytes (56.0 ± 3.46 A.U., *p* < 0.05 vs. CRH; Figure 5H), restoring ROS levels to values not significantly different from those of control oocytes (ns; Figure 5H). These findings indicate that MT protects porcine oocytes from CRH-induced damage by reducing intracellular oxidative stress and inhibiting cumulus cell apoptosis, thereby preserving the developmental competence of the COC.

### 3.6. Porcine Oocytes and Cumulus Cells Express MT1 but Not MT2 Transcripts

**Figure 6:**
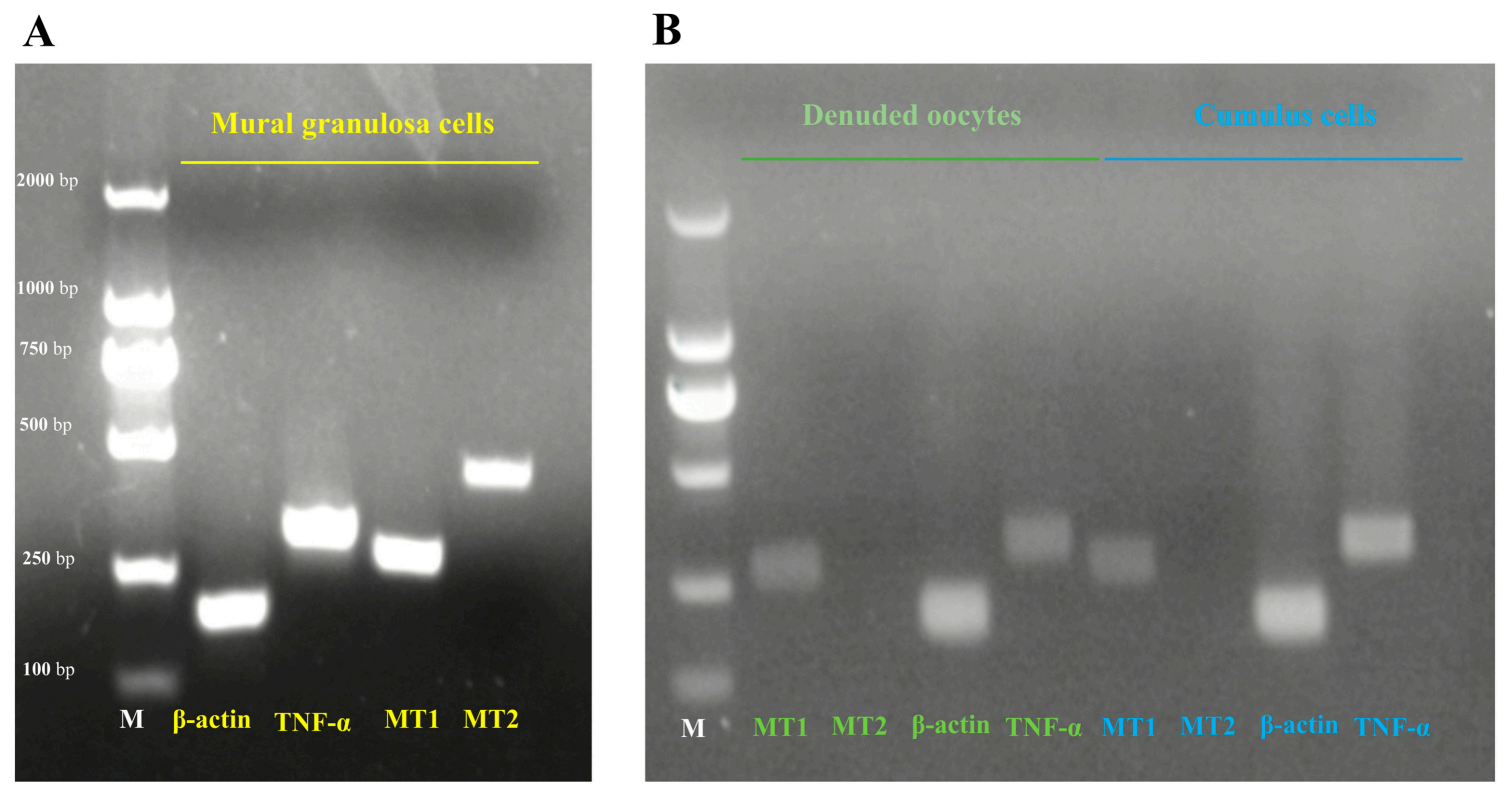
Expression of melatonin receptor subtypes *MT1* and *MT2* in porcine oocytes and cumulus cells. The mRNA expression of *MT1*, *MT2*, and the reference/positive-control genes *β-actin* and *TNF-α* was examined by RT-PCR. (**A**) Porcine mural granulosa cells, which express both *MT1* and *MT2*, served as a positive control to validate primer specificity; distinct bands for *β-actin*, *TNF-α*, *MT1*, and *MT2* were detected. (**B**) Denuded oocytes and cumulus cells both expressed *MT1*, whereas *MT2* transcripts were undetectable in either cell type; *β-actin* and *TNF-α* served as the internal reference and additional positive controls, respectively. M, DNA molecular weight marker.

To identify the melatonin receptor subtype(s) mediating MT’s effects on porcine COCs, the mRNA expression of *MT1* and *MT2* was examined by RT-PCR in DOs and CCs (Figure 6). MGCs were used as a positive control to validate primer specificity. As shown in Figure 6A, distinct bands for *β-actin*, *TNF-α*, *MT1*, and *MT2* were detected in MGCs. In contrast, both DOs and CCs expressed *MT1* mRNA, whereas *MT2* transcripts were undetectable in either cell type (Figure 6B). These results suggest a potential involvement of the MT1 receptor in MT’s protective effects in porcine oocytes and cumulus cells.

## 4. Discussion

Stress exerts its reproductive damage largely through a common downstream pathway. In restraint and other psychological stress models, excessive ROS is generated in ovarian cells, and oxidative stress is increasingly recognized as a primary mechanism by which stress compromises oocytes [6,8]. Although antioxidant protection of oocytes has been reported under various conditions, whether antioxidants differ in their capacity to mitigate stress-induced oocyte damage, and through which mechanisms, remains unclear. The present study addressed this question using two complementary models—an in vivo restraint stress model and an in vitro porcine IVM model supplemented with CRH—both of which reproduced the expected impairment of oocyte development. These two models involve different species and experimental designs, and their findings should be interpreted as complementary, independent lines of evidence rather than a causally integrated, validated unified mechanism. In the mouse model, MT attenuated the systemic stress response; in the porcine model, MT directly protected COCs from CRH-induced damage through antioxidant and anti-apoptotic effects. 

In the in vivo restraint stress model, the advantage of MT was most evident at the endocrine level. Both MT and EGCG improved oocyte development at appropriate doses, but MT did so across a broader dose range and more completely. Importantly, MT reduced serum CRH and cortisol concentrations, whereas EGCG did not, indicating that MT acts on the stress response itself rather than only on its downstream products. It should be noted that corticosterone is the primary glucocorticoid in mice; therefore, the cortisol data in this study should be interpreted as a supportive indicator of HPA-axis activation rather than a definitive measure of total glucocorticoid output. Several complementary mechanisms may underlie this hormone-lowering action. Accumulating evidence indicates that MT acts at multiple levels of the HPA axis: in the hypothalamus, it has been reported to suppress CRH synthesis and release by paraventricular neurons; in the pituitary, MT1 receptor activation inhibits CRH-induced ACTH production through a BMP-4-dependent pathway [33]; and in the adrenal cortex, functional MT1 receptors can directly inhibit ACTH-stimulated cortisol synthesis [34,35,36]. In addition, MT may dampen HPA output through GABAergic signaling in the hypothalamus, and it maintains reciprocal circadian regulation with glucocorticoids—chronic stress suppresses endogenous melatonin secretion, whereas exogenous melatonin restores HPA feedback [37,38]. Finally, because ROS themselves can activate PVN neurons, the antioxidant action of MT may also reduce HPA drive indirectly. 

In contrast, EGCG, the major catechin of green tea, is an exogenous dietary polyphenol that acts mainly through direct chemical antioxidant actions—quenching free radicals and chelating transition metals [39]—and through cell-survival signaling rather than through dedicated endocrine receptors expressed along the HPA axis. Unlike MT, EGCG therefore does not directly target the endocrine limb of the stress response; consistent with its low bioavailability and the narrow, pro-oxidant-prone dose window [40], EGCG partially improved development but, in the present restraint model, did not lower serum CRH or cortisol, and the highest dose (100 mg/kg) even reduced blastocyst formation.

The in vitro porcine CRH model, in which systemic endocrine influences are excluded, allowed the direct cellular action of MT to be examined independently of HPA-axis modulation. MT still rescued MII maturation and blastocyst formation, reduced intracellular ROS, and inhibited cumulus cell apoptosis, demonstrating that MT protects the COC through a local action that does not require systemic endocrine effects. A mechanistic basis for this direct protection is that MT is an endogenous constituent of the ovarian follicle rather than a xenobiotic. It is concentrated in follicular fluid at levels exceeding those in serum [41], and it is locally synthesized by granulosa, cumulus, and oocyte cells that express the melatonin-synthesizing enzymes AANAT and ASMT [42]. The mitochondria themselves are a major site of this synthesis: AANAT co-localizes with mitochondria, and isolated oocyte and cumulus-cell mitochondria release melatonin [43], which in turn enhances respiratory-chain activity, mitochondrial membrane potential, biogenesis and mtDNA copy number, and ATP production while lowering ROS [44]. Exogenous MT during IVM therefore reinforces a pre-existing local antioxidant and energy-supporting system rather than introducing a foreign compound, which may underlie its consistent protection over a broad concentration range (10^−9^–10^−5^ M). It should be noted that the MT concentrations used in the in vitro experiments are higher than endogenous physiological levels, which is common practice in IVM systems due to the absence of local melatonin synthesis and the increased oxidative stress in vitro.

This local protection is also receptor-supported: both denuded oocytes and cumulus cells expressed *MT1* but not *MT2* transcripts, and *MT1* signaling upregulates antioxidant enzymes and cell survival [15,43]. *MT1* expression in porcine cumulus and granulosa cells has been reported independently [28,45], supporting a receptor-mediated action in the COC. Kang et al. previously did not detect *MT1* transcripts in denuded porcine oocytes by RT-PCR [28], whereas we observed *MT1* expression in oocytes under the present conditions, with mural granulosa cells serving as a positive control to validate primer specificity. The reason for this discrepancy is currently unclear and warrants further investigation. Our data therefore support *MT1* in both compartments under the present conditions. Together, the in vitro findings indicate that MT acts directly within the COC through an endogenous, mitochondrially integrated and receptor-supported antioxidant pathway—an action distinct from, and complementary to, the systemic endocrine effect demonstrated in vivo.

## 5. Conclusions

In conclusion, using two complementary experimental models, this study provides independent evidence that MT protects oocytes from stress-induced developmental impairment. Systemically, MT uniquely attenuates stress hormone (CRH and cortisol) secretion, reducing the overall stress burden, whereas EGCG lacks this capacity. At the cellular level, MT directly protects porcine oocytes from CRH-induced damage by reducing intracellular ROS accumulation and inhibiting cumulus cell apoptosis. Furthermore, RT-PCR analysis revealed exclusive transcript expression of *MT1* (but not *MT2*) in porcine oocytes and cumulus cells, suggesting a potential involvement of the MT1 receptor in mediating these protective effects. These findings establish MT as a superior antioxidant for mitigating stress-related reproductive compromise and identify MT1 as a candidate molecular target, providing a mechanistic foundation for the therapeutic application of MT in both clinical reproductive medicine and livestock production.

## Figures and Tables

**Table 1:** Oligonucleotide primer sequences used for real-time PCR in this study.

cDNA	Gene ID	Oligonucleotide Sequences (5′–3′)	Amplified Product Size (bp)
*β-actin*	414396	F: CGTGCGGGACATCAAGGA R: AGGAAGGAGGGCTGGAAGA	177
*TNF-α*	397086	F: ACCACGCTCTTCTGCCTACTG R: GACGGGCTTATCTGAGGTTTGAGA	351
*MT1*	100627802	F: ATACGACAGGTGGTACAGCA R: ACTGACGGACTGTGCAAACGT	282
*MT2*	100511397	F: CGGTCGTGTGCTTCTGTTACC R: AGCAGACGGCGAAGATCA	406

**Table 2:** Effects of melatonin and EGCG supplementation during in vitro maturation on developmental competence of oocytes from mice subjected to 48 h restraint stress.

Mouse Treatments			Oocytes Cultured	Mean ± SEM % of 2-Cell (Activated Oocytes)	Mean ± SEM % of 4-Cell (2-Cell Stage)	*p*-Value	Mean ± SEM % of Blastocysts (4-Cell Stage)	*p*-Value
Restraint	MT (M)	EGCG (μg/mL)						
−	−	−	130	96.1 ± 1.4	78.9 ± 1.4		44.9 ± 1.9	
+	−	−	286	95.1 ± 0.8	58.2 ± 2.5 ^##^	0.000354	20.8 ± 2.1 ^##^	0.000483
+	1 × 10^−9^	−	101	95.7 ± 2.9	53.2 ± 6.7	0.728	27.1 ± 6.1	0.396
+	1 × 10^−7^	−	124	97.1 ± 2.1	57.4 ± 6.5	0.702	24.5 ± 4.7	0.582
+	1 × 10^−5^	−	158	96.5 ± 2.3	71.2 ± 7.2 *	0.012	46.9 ± 7.3 **	0.000204
+	−	10	146	94.9 ± 0.7	67.1 ± 5.9	0.057	26.9 ± 8.1	0.326
+	−	20	126	90.6 ± 6.6	72.2 ± 6.6 *	0.013	28.5 ± 5.1	0.255

−, no addition or no treatment; +, addition or treatment. Two-cell rate = percentage of 2-cell embryos relative to activated oocytes; 4-cell rate = percentage of 4-cell embryos relative to 2-cell stage embryos; blastocyst rate = percentage of blastocysts relative to 4-cell stage embryos. Data are presented as mean ± SEM. Data were analyzed by one-way ANOVA followed by Dunnett’s post hoc test. ^##^ *p* < 0.01 vs. control group (no restraint, no drug treatment); * *p* < 0.05, ** *p* < 0.01 vs. restraint group (restraint stress only, no drug treatment).

## Data Availability

The original contributions presented in this study are included in the article/Supplementary Materials. Further inquiries can be directed to the corresponding author.

## Supplementary Materials

The following supporting information can be downloaded at: https://www.mdpi.com/article/10.3390/ani16213403/s1, Figure S1: Confirmation of germinal vesicle (GV) stage in mouse and porcine oocytes; Figure S2: Morphological identification of porcine mural granulosa cells during isolation.

## Abbreviations

The following abbreviations are used in this manuscript:

ACTH	Adrenocorticotropic hormone
A.U.	Arbitrary units
BMP-4	Bone morphogenetic protein-4
CRH	Corticotropin-releasing hormone
CBG	Corticosteroid-binding globulin
COCs	Cumulus-oocyte complexes
DOs	Denuded oocytes
EGCG	Epigallocatechin gallate
GABA	γ-aminobutyric acid
GV	Germinal vesicle
HPA	Hypothalamic–pituitary–adrenal
IVM	In vitro maturation
i.p.	Intraperitoneal
MT	Melatonin
MT1	Melatonin receptor 1 (MTNR1A)
MT2	Melatonin receptor 2 (MTNR1B)
MII	Metaphase II
MGCs	Mural granulosa cells
PA	Parthenogenetic activation
PB1	First polar body
ROS	Reactive oxygen species
TNF-α	Tumor necrosis factor-α
TUNEL	Terminal deoxynucleotidyl transferase dUTP nick end labeling
