# Supplementary material for: Melatonin Ameliorates Stress-Induced Oocyte Developmental Impairment: Evidence from In Vivo and In Vitro Models
Source: Animals (Basel). 2026 Oct 28;16(21):3403. doi: 10.3390/ani16213403 (PMC13762362; doi:10.3390/ani16213403)
Supplement: Supplementary file 1 [file animals-16-03403-s001.zip › animals-4609977-supplementary.pdf]

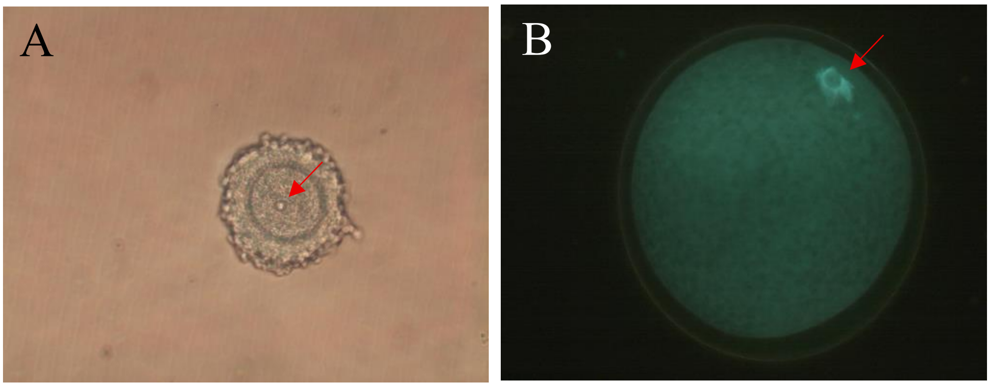

Figure S1. Confirmation of germinal vesicle (GV) stage in mouse and porcine oocytes. (A) Mouse COC collected at 48 h after eCG injection. The GV is directly visible under a stereomicroscope as a large, clear nucleus in the center of the oocyte (red arrow). No polar body was observed, consistent with the GV stage. (B) Porcine oocyte stained with Hoechst 33342. Due to the abundant cytoplasmic lipid droplets in porcine oocytes, the GV cannot be directly observed under bright-field microscopy; therefore, Hoechst staining was performed to visualize the germinal vesicle (red arrow indicates the bright fluorescent region of the GV). COCs aspirated from 3–6 mm antral follicles were confirmed to be at the GV stage by this method.

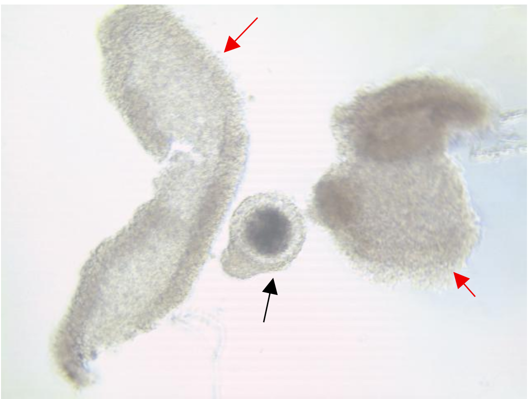

Figure S2. Morphological identification of porcine mural granulosa cells during isolation. Representative stereomicroscopic image of cell populations in sedimented follicular fluid. Sheet-like aggregates of mural granulosa cells (MGCs, red arrows) were selectively collected as the target cell population based on their characteristic morphology, while intact cumulus-oocyte complexes (COCs, black arrow) were excluded during the MGC isolation procedure.
